# CARIBOU‐1: A pilot controlled trial of an Integrated Care Pathway for the treatment of depression in adolescents

**DOI:** 10.1002/jcv2.12083

**Published:** 2022-05-27

**Authors:** Darren B. Courtney, Amy Cheung, Joanna Henderson, Kathryn Bennett, Wei Wang, Sheng Chen, Marco Battaglia, John Strauss, Rachel Mitchell, Karen Wang, Jacqueline Relihan, Matthew Prebeg, Karleigh Darnay, Peter Szatmari

**Affiliations:** ^1^ Department of Psychiatry University of Toronto Toronto Ontario Canada; ^2^ Department of Clinical Epidemology & Biostatistics McMaster University Hamilton Ontario Canada; ^3^ Centre for Addiction and Mental Health Toronto Ontario Canada; ^4^ Island Health Nanaimo Ontario Canada; ^5^ Margaret and Wallace McCain Centre for Child, Youth and Family Mental Health Toronto Ontario Canada

**Keywords:** adolescent, care pathway, depression, pilot controlled trial

## Abstract

**Background:**

To co‐ordinate a multidisciplinary team in the delivery of guideline recommendations using a measurement‐based care framework, our group previously developed a care pathway for the treatment of depression in adolescents. Core components of the pathway were: assessment, education, cognitive‐behavioural therapy, a caregiver intervention group, a medication algorithm, and monthly measurement‐based care “team reviews” with the adolescent present. The aim of this study was to test the feasibility of conducting a controlled clinical trial of the pathway.

**Method:**

We conducted a 20‐week pilot controlled clinical trial of the care pathway relative to treatment as usual. Participants were adolescents (age 14–18) with a primary diagnosis of Major Depressive Disorder recruited from one of two outpatient psychiatric clinics at academic hospitals. Site of presentation was the method of allocation. Thirty‐five youth were allocated to the pathway and 31 were allocated to treatment as usual. As this is a pilot study, trial feasibility outcomes were of primary interest, including clinician fidelity to the care pathway.

**Results:**

Our target sample size was recruited over a 15‐month time interval. Clinician fidelity and adolescent engagement in the care pathway components on a priori checklists were high (95% and 80%, respectively). We collected baseline and 20‐week endpoint data for our primary outcome of the Children's Depression Rating Scale – Revised (CDRS‐R) for 83% of the sample. On linear mixed effects modelling, we observed a linear decrease in CDRS‐R across 4‐week intervals up to the 20‐week endpoint in both groups (*β* = −2.07; 95% CI −3.14 to −1.01).

**Conclusion:**

A controlled clinical trial of a complex, multi‐component intervention for the treatment of depression in adolescents is feasible. Given the need to find optimal strategies to deliver effective care for adolescents with depression, a definitive randomized controlled trial of the pathway is warranted.

Trial is registered at Clinicaltrials.gov: NCT03428555

## Graphical abstract

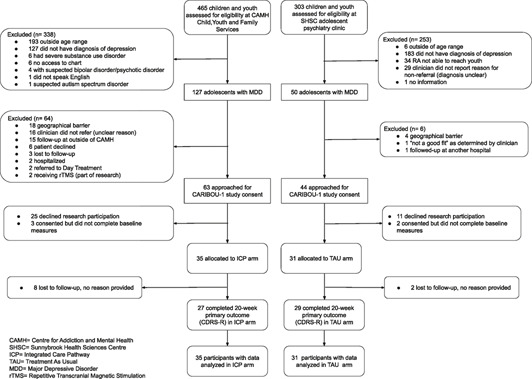

We conducted a pilot controlled clinical trial for the treatment of depression in adolescents comparing a care pathway relative to treatment‐as‐usual. The trial demonstrated feasibility with respect to recruitment, adherence to the pathway and data collection efficiency.

**Key points**

- The NICE guideline for have been appraised as a high quality guideline for the treatment of depression in adolescents
- Many adolescents with depression do not receive guideline‐adherent care
- It is feasible to implement and test a collaboratively developed Integrated Care Pathway for the treatment of adolescent depression relative to treatment as usual to facilitate the delivery of guideline‐adherent care

Major Depressive Disorder in adolescents (MDD‐A) is common (Avenevoli et al., 2015; Cheung & Dewa, 2006; Polanczyk et al., 2015), debilitating (Clayborne et al., 2019; Fergusson & Woodward, 2002; Gore et al., 2011) and a risk factor for suicide (Renaud et al., 2008). Treatment approaches used in real‐world clinical practice are heterogeneous, and often insufficiently supported by evidence (Watson et al., 2019). Greater adherence to care standards in the treatment of depressive disorders in adolescents treated in primary care has been associated with improved outcomes (Wells et al., 2012). Circumscribed evidence‐based treatment approaches like antidepressant medication and/or cognitive behavioural therapy (CBT) have had limited benefits, with up to 40% of adolescents unremitted at follow‐up (March et al., 2009; Vitiello et al., 2011; Cuijpers et al., 2021). High‐quality clinical practice guidelines, like the NICE guideline for depression in children and young people (Bennett et al., 2018; NICE, 2019), call for more comprehensive care in addition to medications and therapy, including: structured assessment; education about sleep, exercise and diet; family involvement and; measurement‐based care (MBC). While other research groups have explored service delivery models (Asarnow et al., 2005; Martinez et al., 2018) and MBC strategies (Abright & Grudnikoff, 2020; Bickman et al., 2011; Chorpita et al., 2017; Emslie et al., 2004; Gunlicks‐Stoessel et al., 2019), the effectiveness of Integrated Care Pathways (ICPs) to guide comprehensive delivery of care for MDD‐A has not been studied. ICPs are pre‐defined treatment flow processes intended to co‐ordinate the use of clinical practice guideline recommendations for a given condition (Campbell et al., 1998). They have the potential to help both the standardization and personalization of management of a given condition by facilitating the delivery of comprehensive and multidisciplinary care through a series of decision points and treatment steps. The ultimate aim of ICPs is to optimize the provision of evidence‐based care through finding the intersection between research evidence, clinical judgement of the expert provider and patient/family values in the context of finite resources (Courtney, Bernett, & Szatmari, 2019; Sackett et al., 1996; Allen et al., 2009).

Our group collaboratively and systematically developed an ICP for the treatment of adolescent depression (Courtney, Bennett, Henderson, et al., 2019) based on a contextualization of the NICE guideline recommendations (NICE, 2019). The resulting ICP has been called the CARIBOU‐1 pathway, representing “Care for Adolescents who Receive Information ‘Bout OUtcomes” (first iteration). The components of the CARIBOU‐1 pathway offered to youth include: (1) a structured assessment, with particular attention to assessment of risk for self‐harm, exposure to bullying and caregiver mental illness, (2) a group education session (called Mood Foundations) where the nature of depression and the role of sleep hygiene, exercise and diet are discussed with youth and caregivers, (3) 16 sessions of group CBT (group therapy was offered rather than individual therapy, reflecting the available resources at our centre and some evidence of similar efficacy between the two CBT formats (Rosselló et al., 2008)), (4) an 8‐session caregiver group focussing on validating communication and collaborative problem‐solving, (5) a medication algorithm with fluoxetine as the first‐line treatment and sertraline as second‐line, (6) all in the framework of MBC. MBC took place through monthly “Team Reviews” whereby youth meet with their main clinician (most often a psychiatrist), any other clinicians involved (often a social worker co‐facilitating the CBT group) and, at the youth's discretion, the youth's caregiver (e.g. parent). At these Team Reviews, participants reviewed changes in scale scores on measures of depressive symptoms (youth reported Mood and Feelings Questionnaire (Angold & Costello, 1987; MFQ), general functioning (Columbia Impairment Scale – youth and caregiver versions; YCIS & PCIS; completed by the relevant informant (Bird et al., 1993)) and family functioning (McMaster Family Assessment Device general functioning subscale – MFAD‐GF (Epstein et al., 1983) completed separately by youth and caregivers). Results from these measures were used to collaboratively decide on starting, continuing, intensifying, switching, tapering or stopping various treatment components. This approach makes the pathway both standardized and personalized. More details of the pathway are well documented elsewhere (Courtney, Bennett, Henderson, et al., 2019; Courtney, Cheung, et al., 2019; Courtney & Szatmari, 2020).

The literature supports the concept of patient engagement in research to improve outcomes (Domecq et al., 2014; O’Mara‐Eves et al., 2015). The Youth Engagement Initiative (YEI) (Heffernan et al., 2017) at our centre is a team of youth engagement facilitators on staff who are young people with lived/living experience of mental health and substance use challenges and work with a broader network of paid youth advisors (e.g. National Youth Action Council (Mood Disorders Society of Canada, n.d.) for purposes of making research innovations relevant to the people they are intended to serve. Investigators worked closely with the YEI to develop materials related to recruitment, orientation, and treatment components as they pertained to the CARIBOU‐1 intervention and pilot study (Cundill Centre for Child and Youth Depression, n.d.).

The current study's aim is to examine the feasibility of a controlled clinical trial testing the effectiveness of the CARIBOU‐1 pathway on reducing depression symptom severity over 20 weeks relative to treatment as usual (TAU) for adolescents with MDD‐A. Our main hypotheses and benchmarks for a successful pilot study were that:

(1) At least 30 participants at each of two sites over a span of 21‐month would be recruited.
(2) 95 percent of youth participants would be able to complete the baseline assessment within a span of 2 hours.
(3) Within the ICP arm, clinicians would be able to achieve 90% adherence to the ICP model as per a prespecified checklist.
(4) As a benchmark of retention, 80% of the ideal scheduled data points on the primary clinical outcome (Children's Depression Rating Scale‐ Revised), would be obtained.

## METHODS

Our methods are detailed in our published protocol, with few deviations (each are mentioned below) (Courtney, Cheung, et al., 2019). In brief, this was a pilot parallel non‐randomized controlled clinical trial. Psychiatrists assessed adolescents aged 14–18 presenting to the outpatient psychiatry clinics at one of two academic hospitals in Toronto, Ontario, Canada: the Centre for Addiction and Mental Health (CAMH) and Sunnybrook Health Sciences Centre (SHSC). SHSC also ran a satellite clinic in the community where youth are assessed by a psychiatrist and treated. If depression was identified as a primary presenting concern for consenting youth, a research analyst conducted an assessment for inclusion/exclusion criteria.

### Ethical considerations

The study was approved by the CAMH and SHSC research ethics boards.

### Inclusion and exclusion criteria

Youth were included into the trial if they met criteria for MDD based on DSM‐5 criteria as assessed by a semi‐structured diagnostic interview (the Diagnostic Interview for Affective Symptoms for Children – DIAS‐C (Merikangas et al., 2014)). Youth were excluded if they presented with active and impairing psychotic symptoms, bipolar disorder, moderate to severe substance use disorder, moderate to severe eating disorder, autism spectrum disorder, intellectual disability, or imminent risk of suicide requiring hospitalization. Caregivers were included if the enrolled youth agreed to caregiver involvement in the trial. Youth and caregivers also needed to be able to read and write in English and provide informed consent.

### Treatment allocation

Youth were allocated to one of two treatment models based on site of presentation. The intervention is applied at the clinic level, so randomizing participants to the treatment within site was not possible without the risk of contamination effects. Enrolled youth presenting to CAMH were assigned the CARIBOU‐1 pathway, while those presenting to SHSC were assigned to TAU.

### Clinical research outcome measures

These were collected at baseline and every 4 weeks for 20 weeks including the evaluator‐rated Children's Depression Rating Scale – Revised (CDRS‐R; Poznanski et al., 1985), self‐rated WHO Disability Assessment Scale – Child/Youth version 2.0 (WHODAS CY 2.0; Scorza et al., 2013) and caregiver‐rated Child Behaviour Checklist internalizing subscale (CBCL‐int; Achenbach & Edelbrock, 1991). The Childhood Interview for Borderline Personality Disorder (CI‐BPD; Sharp et al., 2012) and Beck Hopelessness Scale (BHS: Beck & Steer, 1988) were collected at baseline to further describe the sample. The psychometric properties of these measures are detailed in our protocol (Courtney, Cheung, et al., 2019). These research outcomes were not provided to clinicians and youth participants to maintain some independence from the MBC‐framework of the intervention.

### Clinician adherence and youth engagement in the pathway

Clinician adherence was obtained using the a priori determined checklist (see Appendix of the protocol (Courtney, Cheung, et al., 2019)) with one deviation from the original protocol; that is, the component criteria “team review offered every 4 weeks” was omitted from the checklist scores as the concept was found to be too broad to operationalize and was better captured in subsequent more detailed criteria around receipt of MBC. For each youth study participant, clinician adherence was calculated as the number of components the clinician offered divided by the number of components that were applicable for that youth x 100%. During the data collection phase, we also created a form matching the clinician adherence checklist to extract and code youth engagement in each of the components.

### Inter‐rater reliability

The inter‐rater reliability for semi‐structured interviews pertaining to evaluator‐rated measures was calculated by having the principal investigator (DBC) and the research assistant independently rate audio recordings; 6 for the DIAS‐C, 10 for the CDRS‐R and 6 for the CI‐BPD. All six participants with audio‐recorded DIAS‐C diagnostic assessments were coded as meeting criteria for MDD‐A, and perfect agreement was achieved with regards to this classification. With respect to the CDRS‐R, weighted kappas ranged from 0.77 to 0.93. With respect to the CI‐BPD, weighted kappas ranged from 0.63 to 0.83 on five ratings, with one early participant's recording removed as it was an outlier (weight kappa = −0.42). Weighted kappas are the reported statistic here as item‐level scores are ordinal in nature; greater between‐rater score differences on an item are given more weight than smaller differences.

### Statistical analysis

Baseline differences between groups were calculated using chi‐square or Fisher's exact tests for binary data, Mann–Whitney U tests for non‐parametric continuous data and Student's *t*‐test for parametric data. Feasibility outcomes were assessed as count and proportion data. Clinician adherence and participant engagement percentage scores were summed and divided by the number of youth to provide the mean score. Differences in exposure to various treatment components were calculated using chi‐square analysis, or Fisher exact tests if one cell had a value of ≤5. None of these comparisons were testing a priori hypotheses (i.e. exploratory); therefore, significance threshold was set at *p* ≤ .05 (two‐tailed). The above analyses were conducted using Stata software version 17 (2021).

Linear mixed effects modelling was used to describe changes in clinical research outcome measure scores (i.e. CDRS‐R, WHODAS CY 2.0 and CBCL‐int) over time within groups. Baseline score for the variable of interest, time, and treatment arm were included as the fixed effects. The random participant effect was also included in the model to account for intra‐participant correlation over time. In a sensitivity analysis, a time × time interaction term was added to the model to investigate the possibility of a quadratic pattern to the data, as decelerating patterns of change had been found in other studies (Varigonda et al., 2016). In a further analysis, we also added the treatment × time term the initial model. In a large trial, this treatment × time interaction term from the models would be the term of interest. We calculated the treatment × time interaction here for description purposes with two notable caveats; (1) Pilot studies are not intended to be hypothesis‐testing with respect to clinical outcomes (Thabane et al., 2010), particularly as the sample size would not have sufficient power; (2) The intra‐class correlation with respect to the CDRS‐R over time was relatively high, at 0.119, indicating that site‐level effects are too strong to make appropriate comparisons between sites. An intent‐to‐treat approach was used. Multiple imputation, with 20 imputed sets, was used to address missing data. Regression analyses were conducted using SAS software version 7.1 (2014).

Within the ICP arm, mixed effects modelling was also used to describe the within group changes in the measurement‐based care measure scores over time, as a reference for other groups considering implementing service delivery models for the treatment of adolescent depression. Baseline score for the variable of interest and time terms were included as the fixed effects for this model, with random participant effect included here again.

## RESULTS

### Recruitment and sample characteristics

One hundred and seven youth diagnosed with major depressive disorder were approached by the research analyst. Of these, 66 participants were enrolled in the study: 35 in the CARIBOU‐1 intervention group over a 13‐month time span (March 2018 to April 2019) and 31 in the TAU group over a 15‐month time span (December 2018 to March 2020). Sample ascertainment is described in the CONSORT Flow Diagram in Supplementary Figure S1. 10 youth recruited from the SHSC sample were recruited from the community satellite clinic. Baseline characteristics are described in Supplementary Table S1. The median age of the sample was 16 years old. The sample was predominantly white (53%; 35 of 66) and predominantly cis‐gender girls (68%; 45 of 66). The household income was significantly higher in the TAU group. The median age of onset for MDD was numerically lower in the CARIBOU‐1 intervention group (*p* = .06). Most of the overall sample (80%; 53 of 66) met criteria for comorbid generalized anxiety disorder. One participant met criteria for oppositional defiant disorder, and none met criteria for conduct disorder. The participants in the CARIBOU‐1 intervention group had significantly higher scores on the CDRS‐R and CBCL “withdrawn/depressed” subscale at baseline relative to TAU, representing greater severity of depressive symptoms. Sixty‐three percent (42 of 66, with no significant difference between groups) were taking an antidepressant at baseline.

### Reach and examination of ascertainment bias of sample

Supplementary Figure S1 outlines the ascertainment of the sample at CAMH during recruitment period (March 2018 to April 2019). Of youth who were approached to engage in the study at the CARIBOU‐1 intervention site (CAMH, *N* = 63), we compared those who enrolled in the study (*n* = 35) and those who declined (*N* = 28) with respect to four variables of interest at intake. We did not observe significant differences between those who enrolled and those who declined the research study with respect to age (Mann–Whitney U, *z* = 0.55, *p* = .58), proportion identifying as girls (*χ* ^2^ = 0.01, *p* = .94), socioeconomic status (Mann–Whitney U, *z* = 0.51, *p* = .61) and Patient Health Questionnaire‐9‐Adolescent scores at intake (Mann Whitney U, *z* = −1.31, *p* = .19).

### Clinician adherence and youth participant engagement for the CARIBOU‐1 intervention

Within the CARIBOU‐1 intervention arm, mean clinician adherence to the pathway was 95% (SD 9%). Mean participant engagement was 80% (SD 18%). Supplementary Table S2 outlines clinician adherence and participant engagement in pathway‐specific components (i.e. Mood Foundations, the Caregiver Group and Measurement‐Based Care). Supplementary Tables S3 and S4 outline adherence/engagement outcomes for psychotherapy components and medication components, respectively.

### Differential exposure between CARIBOU‐1 relative to treatment‐as‐usual

In the CARIBOU‐1 group, 26 youth had documentation of MBC, whereas 3 had documentation of receiving MBC in the TAU group (of 29 where charts were available for review). This difference was significant (*p* < .001). In the CARIBOU‐1 group, 20 youth were documented to have received at least one session of group CBT, relative to none in the TAU group (*p* < .001). Twenty‐five and 24 youth in the CARIBOU‐1 and TAU group, respectively, received at least one session of any psychotherapy during the trial (*p* = .95). In the TAU group, 3 were documented to have received individual CBT, 1 interpersonal therapy, 1 dialectical behaviour therapy skills group, 1 family therapy, 1 had therapy in the context of day hospital and 16 were documented to have received an individual therapy, but the type of therapy was not specified. Differential exposure to medications is outlined in Supplementary Table S4.

### Data collection efficiency and participant retention

The median time to complete the baseline assessment was 54 min (IQR: 45–72 min, Range: 31–158 min). Most participants (95.5%) completed the baseline assessment in under 2 hours. With 66 participants, each with 6 potential longitudinal data points on the primary outcome (CDRS‐R), there were 396 ideal data points collected for this measure. We collected 308 data points on the CDRS‐R, indicating a data collection‐efficiency rate of 78%. Fifty‐five of the 66 participants (83%) who provided a baseline primary outcome measure (CDRS‐R) also provided a 20‐week primary outcome measure.

### Description of course of clinical research outcome measures (*n* = 66)

The linear mixed effects model of CDRS‐R scores found a main effect of time of *β* = −2.07 (95% CI −3.14 to −1.01); that is, a decrease of 2.07 points on the CDRS‐R every 4 weeks was estimated. When the treatment x time term was added to the model, the regression co‐efficient for this interaction was *β* = −.008 (95% CI −2.01 to 1.99). With respect to the WHODAS CY 2.0, the main time effect was represented as *β* = −1.43 (95% CI −2.3 to −0.55). When added, the treatment × time interaction term was *β* = 1.34 (95% CI −0.42 to 3.10). The model of CBCL‐int scores found a main time effect of *β* = −1.41, 95% CI −1.94 to −0.86). When added, the treatment × time interaction term had a regression co‐efficient of *β* = −.38, 95% CI −1.47 to 0.72).

These models did not demonstrate significant time × time interaction term on the CDRS‐R, WHODAS or CBCL internalizing subscale scores; that is, we did not find evidence of a quadratic pattern to the data over time.

### Description of course of MBC measures in the CARIBOU‐1 intervention arm (*n* = 35)

Through an exploratory linear mixed effects modelling analysis on available MBC measurement data (non‐imputed), we found that the MFQ scores decreased by 1.05 points (95% CI of −2.05 to −0.04), YCIS scores decreased by 0.93 points (95%CI of −1.40 to −0.46), PCIS scores decreased by 1.31 points (95%CI of −2.05 to −0.56) for every 4 weeks in the pathway. We did not find evidence that MFAD scores changed over time (*β* = .005, 95% CI of −0.038 to 0.029).

## DISCUSSION

This pilot study of the CARIBOU‐1 pathway for the treatment of adolescent depression is, to our knowledge, the first to explore a complex care model, collaboratively developed with youth with lived experience and aligned with appraised high quality clinical practice guidelines and using measurement‐based care. Our primary objective of this pilot was met; that is, to assess feasibility outcomes with respect to conducting a properly powered controlled clinical trial. More specifically, we recruited our target sample size of over 60 participants in a reasonable interval of 15 months. Furthermore, the comprehensive baseline assessment was completed within an acceptable time span of 2 hours. We also collected primary clinical outcome data at nearly 80% of our ideal time points, with over 80% completing the 20‐week endpoint primary outcome measure (i.e. the CDRS‐R). Clinician fidelity to the CARIBOU‐1 pathway model was also high at 95% on an a priori locally developed checklist.

We suspect that the success of these feasibility outcomes are, in part, due to the input of the Youth Engagement Initiative at CAMH (Heffernan et al., 2017). Youth partners were involved in creating materials used to describe the pathway, reviewing accessible language in consent forms, and provided consultation with respect to how to approach youth for the study. They also provided substantial input into the content and design of the materials used for the intervention itself (Courtney, Bennett, Henderson, et al., 2019).

Multi‐disciplinary clinicians were involved in the creation of the pathway (Courtney, Bennett, Henderson, et al., 2019), which we believe contributed to good fidelity. Youth engagement with pathway components was good at 80%. Indicated caregiver engagement in the caregiver‐specific group was low at 42%. Qualitative analysis of interviews and focus groups is planned to learn about ways to further improve clinician fidelity and youth participant engagement in treatment for next iterations of the pathway.

At CAMH, the reach of the pathway was limited by geographical barriers as frequent in‐person group sessions and team reviews required proximity to the hospital site. Strategies to overcome these barriers, such as internet‐based services, are worth exploration.

The participant samples obtained by our recruitment procedures were characterized as being predominantly cis‐girls, consistent with the gender distribution of depression of adolescents in community samples (Cheung & Dewa, 2006; Mojtabai et al., 2016). About half of the sample identified as white, with the other half from various racial backgrounds, representative of Toronto's diversity (City of Toronto, 2021). The majority of participants in our sample endorsed threshold criteria for generalized anxiety disorder (80%), which is a higher rate than in the IMPACT trial (21%; Goodyer et al., 2017) and all anxiety disorders in samples of depressed adolescent studied in large controlled trials (e.g. any anxiety disorder in TADS; 27% (March et al., 2004) and in TORDIA; 36% (Brent et al., 2008)). Conversely, our sample also had very few participants who endorsed criteria for oppositional defiant disorder or conduct disorder (1.5%), which is much lower that other samples (e.g. TADS 23% (March et al., 2004), TORDIA 10% (Brent et al., 2008), IMPACT 12% (Goodyer et al., 2017)). These differences may be related to specific referral processes. Alternatively, these large RCTs used the Schedule for Affective Disorders and Schizophrenia for School‐Age Children‐Present and Life‐time Version (Kaufman et al., 2000) for their diagnostic assessments, whereas this current study used the DIAS‐C (Merikangas et al., 2014). Questions, prompts and branching logic are different between these two diagnostic assessments, which may affect categorization of diagnoses. Participants receiving CARIBOU‐1, on average, were from significantly lower income levels compared to the TAU arm, which was anticipated given the geographical locations of each site in the city. What was not anticipated was that the CARIBOU‐1 sample would have greater depression symptom severity scores on both the CDRS‐R and the CBCL; this may have been due to chance, to associations with income level or differences in referral patterns at the two sites.

Overall, the sample showed evidence of improvement in both groups in mood symptoms on the CDRS‐R. In contrast, we did not observe improvements in functioning on the WHODAS CY 2.0. With few options of validated measures of function in youth, the WHODAS CY 2.0 was chosen as a measure to have a measure of functioning independent from the measurement‐based care component of the intervention; however, studies examining its validity, reliability and responsiveness are limited. The lack of change observed could be related to lack of responsiveness in the measure or a true lack of change in functioning. In the CARIBOU‐1 intervention arm, we did find significant reductions in both the youth reported and caregiver‐reported versions of the CIS, suggesting the former explanation is more likely. The relatively high ICC and linear modelling of the longitudinal CDRS‐R data (vs. a quadratic model) may inform future trial designs and power calculations.

A future iteration of the pathway is already being planned with two key additions. (1) Given the expected limited engagement and retention in CBT, we will plan to have a second line therapy that is also evidence‐based and feasible in the context of limited resources; namely, “Brief Psychosocial Intervention” (Goodyer et al., 2017). (2) We also want to more formally ensure that principles of shared decision‐making (Charles et al., 1997) are being applied at team reviews. These principles include giving emphasis to the patient's perspective, the clinician offering of at least two treatment options (e.g. starting or not starting medication), and accepting that the patient's decision may differ from clinical recommendations (Zisman‐Ilani et al., 2021). The concept of shared decision‐making has become a formal field of study, though with notable gaps in the field of child and adolescent psychiatry (Hayes et al., 2021). It nicely fits in with the aims of ICPs and MBC by optimizing the chances that patient/caregiver values are being accounted for at the key decision‐points, consistent with our ideal view of evidence‐based care.

A larger RCT of the pathway is underway testing both effectiveness and implementation outcomes (Courtney, Barwick, et al., 2022). In the context of this trial, we will also be able to conduct a detailed examination of the role of baseline variables associated with outcome. Among an extensive list of candidate variables (Courtney, Watson, et al., 2022) are measures relating to sleep (Wang et al., 2016) and exercise (Brand et al., 2017). As the role of technology in the management of depression is increasing in prominence (Marciano et al., 2022; Sequeira et al., 2019, 2020), we are also piloting a remote electronically delivered version of the pathway (Courtney et al., 2021).

### Limitations

Allocation to treatment was not randomized and fidelity to the model was assessed using a chart review checklist as opposed to video recordings of sessions. This study was conducted at academic centres and the results may not apply to community settings.

## CONCLUSION

Our feasibility outcomes are favourable with proceeding with a full‐scale cluster randomized controlled trial. The design and planning phase of a definitive trial is already underway, with materials and pilot data being used to facilitate its execution. Should the results of our definitive trial show that the CARIBOU pathway leads to better clinical outcomes, can be delivered with fidelity, and be cost‐effective, investing in wide‐spread use of the model would be warranted. The broad‐scale delivery of comprehensive, guideline‐adherent, standardized and personalized treatment through an ICP for adolescents with depression has the potential to unlock all the knowledge we have accrued so far in the field, tipping the balance towards better outcomes relative to current practice and previous clinical trials.

## AUTHOR CONTRIBUTIONS

Darren B. Courtney was the principal investigator. Darren B. Courtney, Amy Cheung, Joanna Henderson, Kathryn Bennett, Marco Battaglia, John Strauss, Karen Wang, Jacqueline Relihan, Matthew Prebeg, Karleigh Darnay and Peter Szatmari provided input on trial design, recruitment strategies, outcome measurement, and interpretation of the findings. Wei Wang and Sheng Chen provided statistical support. Darren B. Courtney, Matthew Prebeg, Jacqueline Relihan, Karleigh Darnay and Peter Szatmari co‐developed the intervention materials.

## CONFLICT OF INTEREST

The authors have declared that they have no competing or potential conflicts of interest.

## ETHICS STATEMENT

The study was approved by the CAMH and SHSC research ethics boards.

## Supporting information

Supplementary Material 1

Supplementary Material 2

Supplementary Material 3

Supplementary Material 4

Supplementary Material 5

## Data Availability

Data available on request due to privacy/ethical restrictions.
