# Supplementary material for: CARIBOU‐1: A pilot controlled trial of an Integrated Care Pathway for the treatment of depression in adolescents
Source: JCPP Adv. 2022 May 27;2(2):e12083. doi: 10.1002/jcv2.12083 (PMC10242836; doi:10.1002/jcv2.12083)
Supplement: Supplementary file 1 — Supplementary Material 1 [file JCV2-2-e12083-s004.pdf]

Figure S1: Reach of CARIBOU-1 Pathway at the Centre for Addiction and Mental Health and Participant Ascertainment Into Study

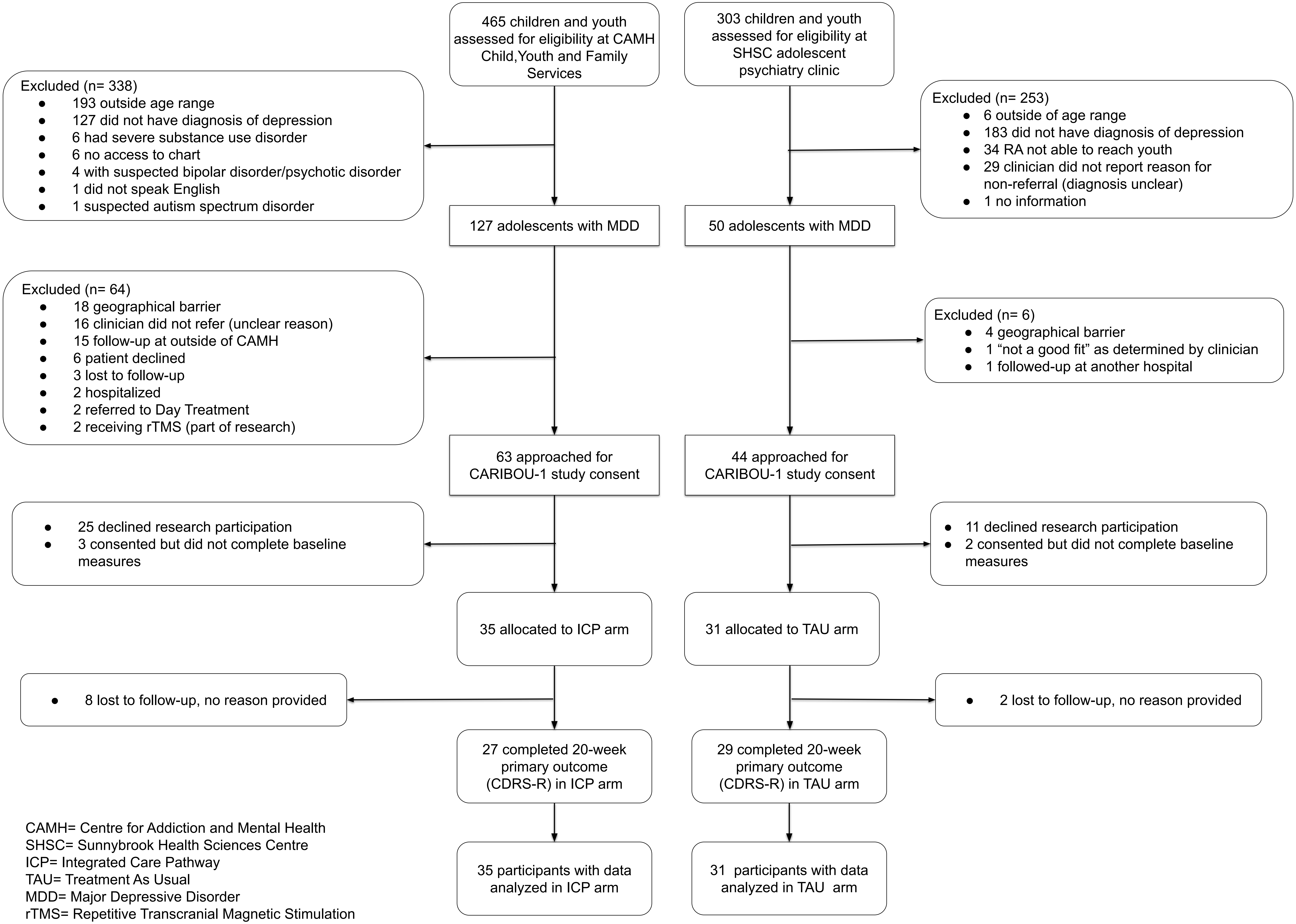

CAMH= Centre for Addiction and Mental Health
SHSC= Sunnybrook Health Sciences Centre
ICP= Integrated Care Pathway
TAU= Treatment As Usual
MDD= Major Depressive Disorder
rTMS= Repetitive Transcranial Magnetic Stimulation
