# Supplementary material for: CARIBOU‐1: A pilot controlled trial of an Integrated Care Pathway for the treatment of depression in adolescents
Source: JCPP Adv. 2022 May 27;2(2):e12083. doi: 10.1002/jcv2.12083 (PMC10242836; doi:10.1002/jcv2.12083)
Supplement: Supplementary file 2 — Supplementary Material 2 [file JCV2-2-e12083-s002.docx]

Table S1: Baseline characteristics of adolescent participants in CARIBOU-1 pilot trial.

| Baseline Variable | CARIBOU-ICP (n=35) | TAU  (n=33) | Statistic | *p*-value |
| --- | --- | --- | --- | --- |
| Age (years) | Median=16  (IQR=15-17;  range 14-18) | Median=16  (IQR=15-17;  range 14-18) | z=-0.855^a^ | 0.39 |
| Gender identity:  Cis-girl  Cis-boy  Nonbinary  Trans-boy | 23  9  3  0 | 22  9  0  2 | χ^2^=0.01^b^ | 0.93 |
| Race:  White  Mixed Race  East Asian/ Southeast Asian  Black or Caribbean  South Asian  Middle Eastern  Latinx | 17  8  4  3  2  0  0 | 18  5  5  3  0  2  1 | χ^2^=0.24^c^ | 0.62 |
| Number of caregivers in home:  Two  One  None | 22  13  0 | 22  5  6 | z=0.248^a^ | 0.80 |
| Household Income  >$150,000  $120,000-$149,999  $90,000-$119,999  $60,000-$89,999  $30,000-$59,999  <$29,000  Did not answer | 7  2  2  4  6  2  12 | 15  2  2  1  0  2  11 | z=-2.61^a^ | <0.01* |
| Age of onset of MDD | Median=13;  IQR=11-15;  Range 5-17) | Median=15;  IQR 12-16;  Range 8-17 | z=-0.186^a^ | 0.06 |
| DIAS-C Diagnosis:  GAD  Social Anxiety Disorder  Panic Disorder  OCD  ADHD  ODD  Conduct Disorder  Mild alcohol use disorder  Mild substance use disorder (non-alcohol, non-tobacco) | 29  16  4  7  10  1  0  0  3 | 24  13  7  5  5  0  0  1  2 | χ^2^=0.31  χ^2^=0.17  χ^2^=1.47  χ^2^=0.21  χ^2^=1.79  exact  exact  exact  exact | 0.60  0.68  0.23  0.64  0.18  NS  NS  NS  NS |
| Borderline Personality Disorder (CIBPD) | 8 | 3 | χ^2^=2.05 | 0.15 |
| Self-Injurious Thoughts and Behaviours  History of suicide attempt  History of non-suicidal self-injury  Item 13 on CDRS-R^d^ | 13  25  Median=1  IQR=1-5;  range 1-7 | 11  16  Median=1  IQR 1-4;  range 1-6 | χ^2^=0.28  χ^2^=2.74  z=0.14^a^ | 0.60  0.10  0.89 |

| Baselines Research Measures:  CDRS-R  WHODAS-2.0-CY total  CBCL-anxious/depressed (t-score)  CBCL-withdrawn/depressed (t-score)  CBCL-somatic(t-score)  CBCL-social problems (t-score)  CBCL-thought problems (t-score)  CBCL-attention problems (t-score)  CBCL-rule-breaking behavior (t-score)  CBCL-aggressive behavior (t-score)  Beck Hopelessness Scale | Median 56  IQR: 46-67;  Range 34-101  Median: 30.7  IQR: 22.1-44.1;  Range: 11.6-59.3  x̄=70.5 (SD=8.4)  x̄=73.6 (SD=9.9)  x̄=63.9 (SD=9.2)  x̄=58.7 (SD=7.9)  x̄=64.3(SD=7.0)  x̄=63.0(SD=9.2)  x̄=56.5(SD=6.2)  x̄=56.4(SD=6.4)  x̄=11.5 (SD=5.4) | Median 49  IQR 29-59;  Range 23-87  Median: 29.5  IQR: 20.3-41.3  Range: 5.4-73.6  x̄=65.0(SD=11.2)  x̄=65.9(SD=8.9)  x̄=61.7 (SD=12.0)  x̄=55.3 (SD=6.7)  x̄=60.8 (SD=6.4)  x̄=60.2(SD=9.1)  x̄=55.6(SD=5.8)  x̄=54.2(SD=4.9)  x̄=12.2 (SD=5.5) | z=2.437^a^  z=-.282^a^  t=1.71  t=2.52  t=0.63  t=1.42  t=1.6  t=0.92  t=0.46  t=1.14  t=-0.48 | 0.015*  0.78  0.09  0.02*  0.53  0.16  0.12  0.36  0.65  0.26  0.63 |
| --- | --- | --- | --- | --- |
| Treatment History at Baseline  Previous trial of CBT, not in CBT at baseline  Previous trial of fluoxetine, not on fluoxetine at baseline  ≥1 previous antidepressant trials  On fluoxetine at baseline  On sertraline at baseline  On any antidepressant at baseline | 2  5  8  14  5  23 | 3  6  8  15  2  19 | exact  χ^2^=2.45  χ^2^=0.08  χ^2^=2.65  exact  χ^2^=0.14 | NS  0.29  0.78  0.27  NS  0.79 |

^a^Mann-Whitney U test used for non-parametric data or ordinal outcome.

^b^Cis-girl relative to not cis-girl

^c^White relative to not white

^d^Item asks about extent of suicidal ideation; scale 1 to 7.

NS= not significant on Fisher’s exact text (*p*>0.025)

*indicates *p*<0.025 (two-tailed threshold for significance, uncorrected for multiple comparisons due to exploratory nature of tests) – NB: No tests in this table would be significant with Bonferroni correction of a threshold at *p*<0.0008.
