# Supplementary material for: CARIBOU‐1: A pilot controlled trial of an Integrated Care Pathway for the treatment of depression in adolescents
Source: JCPP Adv. 2022 May 27;2(2):e12083. doi: 10.1002/jcv2.12083 (PMC10242836; doi:10.1002/jcv2.12083)
Supplement: Supplementary file 3 — Supplementary Material 3 [file JCV2-2-e12083-s003.docx]

Table S2: Clinician Adherence to CARIBOU-1 Intervention Components and Participant Engagement for Mood Foundations, Caregiver Group and Measurement-Based Care

|  | **CARIBOU-1 Pathway** | | | |
| --- | --- | --- | --- | --- |
|  |  | **Clinician Adherence** | | **Participant Exposure** |
| **Component** | **Main Component Participant** | **# applicable** | **# offered (% of applicable)** | **# youth engaged**  **(% of offered)** |
| CARIBOU Mood Foundations Session | Youth (N=35) | 33   - 2 withdrew from pathway just after baseline | 32 (97%)   - 1 participant referred to day program at beginning of pathway | 20 (63%) |
| If either youth-rated or caregiver-rated MFAD ≥2, CARIBOU Caregiver group. | Caregiver (N=22) | 19   - 3 families had MFAD scores<2 - 4 already on sertraline at baseline with some evidence of response | 19 (100%) | 8 (42%)   - 3 caregiver sets on waitlist for group at end of pathway - 8 declined group |
| Any MBC | Youth (N=35) | 26   - 2 withdrew from pathway just after baseline - 1 did not complete measures - 6 missing information | 26 (100%) | 26 (100%) |
| MBC conducted at 4 weeks from baseline | Youth (N=35) | 23   - 2 withdrew - 10 no documentation | 20 (87%)   - 3 “team reviews” documented, but no record of MBC | 16 (80%) |
| MBC conducted at 8 weeks from baseline | Youth (N=35) | 27   - 3 withdrew - 4 no documentation | 25 (93%)   - 2 “team reviews” documented, but no record of MBC | 17 (68%) |
| MBC conducted at 12 weeks from baseline | Youth (N=35) | 23   - 7 withdrew - 3 no documentation - 2 hospitalized | 22 (96%)   - 1 “team review” documented, but no record of MBC | 20 (90%) |
| MBC conducted at 16 weeks from baseline | Youth (N=35) | 23   - 7 withdrew - 3 no documentation - 2 hospitalized | 20 (87%)   - 3 “team reviews” documented, but no record of MBC | 17 (85%) |
| MBC conducted at 20 weeks from baseline | Youth (N=35) | 22   - 8 withdrew - 5 no documentation | 20 (91%)   - 2 “team reviews” documented, but no record of MBC | 19 (95)% |
