# Supplementary material for: CARIBOU‐1: A pilot controlled trial of an Integrated Care Pathway for the treatment of depression in adolescents
Source: JCPP Adv. 2022 May 27;2(2):e12083. doi: 10.1002/jcv2.12083 (PMC10242836; doi:10.1002/jcv2.12083)
Supplement: Supplementary file 4 — Supplementary Material 4 [file JCV2-2-e12083-s001.docx]

Table S3: Clinician Adherence and Participant Engagement to CARIBOU-1 Intervention Components

|  | **CARIBOU-1 Pathway** | | | | **TAU** | | | **Comparison between groups** |
| --- | --- | --- | --- | --- | --- | --- | --- | --- |
|  |  | **Clinician Adherence** | | **Participant Exposure** |  | **Participant Exposure** | | **(exposed relative to applicable)** |
| **Component** | **Main Component Participant** | **# applicable** | **# offered (% of applicable)** | **# youth engaged**  **(% of offered)** | **Main Component Participant** | **# applicable** | **# exposed (% of applicable)** | **p (Fisher’s exact)** |
| Dedicated session for psychoeducation (Mood Foundations) | Youth (N=35) | 33   - 2 withdrew from pathway just after baseline | 32 (97%)   - 1 participant referred to day program at beginning of pathway | 20 (63%) | Youth (N=31) | 29   - 2 missing information | 0 (0%) | <0.001 |
| ≥1 session Group CBT | Youth (N=35) | 31   - 2 withdrew from pathway just after baseline - 2 started CBT group prior to pathway, but dropped out | 30 (97%)   - 1 participant referred to day program at beginning of pathway | 22 (73%) | Youth (N=31) | 29   - 2 missing information | 0 (0%) | <0.001 |
| Completed 16 sessions of Group CBT | Youth (N=35) | 22 | 22 (100%) | 14 (63%)   - 2 were still in group at end point | N/A |  |  | N/A |
| Any psychotherapy |  |  |  |  |  | 29   - 2 missing information | 24 (83%) |  |
| If either youth-rated or caregiver-rated MFAD ≥2, caregiver group. | Caregiver (N=22) | 19   - 3 families had MFAD scores<2 | 19 (100%) | 8 (42%)   - 3 caregivers on waitlist for group at end of pathway | Caregiver |  |  |  |
| If not on antidepressant at baseline, no previous fluoxetine trial and moderate-severe depression, fluoxetine offered as first-line | Youth (N=35) | 19 | 19 (100%) | 19 (100%) | Youth (N=31) |  |  |  |
| If failed fluoxetine, sertraline offered as second-line. | Youth (N=35) | 5 | 3 (60%)   - Escitalopram started by non-study psychiatrists during hospitalization for 2 participants | 3 (100%) | Youth (N=31) |  |  |  |
| If tolerated, medication continued until team review corresponding to “8 weeks since medication initiation” even if no response. | Youth (N=35) | 18 | 18 (100%) | N/A | Youth (N=31) |  |  |  |
| If no response and not remitted at team review corresponding to “12 weeks since medication initiation”, discussion around switching medication. | Youth (N=35) | 6 | 6 (100%) | N/A | Youth (N=31) |  |  |  |
| Not offered St. John’s Wort, Venlafaxine or Tricyclic Antidepressant | Youth (N=35) | 33 | 33 (100%) | 33 (100%) | Youth (N=31) |  |  |  |
| Any MBC | Youth (N=35) |  |  |  | Youth (N=31) |  |  |  |
| MBC conducted at 4 weeks from baseline | Youth (N=35) | 23   - 2 withdrew - 10 no documentation | 20 (87%)   - 3 “team reviews” documented, but no record of MBC | 16 (80%) |  |  |  |  |
| MBC conducted at 8 weeks from baseline | Youth (N=35) | 27   - 3 withdrew - 4 no documentation | 25 (93%)   - 2 “team reviews” documented, but no record of MBC | 17 (68%) |  |  |  |  |
| MBC conducted at 12 weeks from baseline | Youth (N=35) | 23   - 7 withdrew - 3 no documentation - 2 hospitalized | 22 (96%)   - 1 “team review” documented, but no record of MBC | 20 (90%) |  |  |  |  |
| MBC conducted at 16 weeks from baseline | Youth (N=35) | 23   - 7 withdrew - 3 no documentation - 2 hospitalized | 20 (87%)   - 3 “team reviews” documented, but no record of MBC | 17 (85%) |  |  |  |  |
| MBC conducted at 20 weeks from baseline | Youth (N=35) | 22   - 8 withdrew - 5 no documentation | 20 (91%)   - 2 “team reviews” documented, but no record of MBC | 19 (95)% |  |  |  |  |
