# Supplementary material for: CARIBOU‐1: A pilot controlled trial of an Integrated Care Pathway for the treatment of depression in adolescents
Source: JCPP Adv. 2022 May 27;2(2):e12083. doi: 10.1002/jcv2.12083 (PMC10242836; doi:10.1002/jcv2.12083)
Supplement: Supplementary file 5 — Supplementary Material 5 [file JCV2-2-e12083-s005.docx]

Table S4: Clinician Adherence to CARIBOU-1 Intervention Components and Participant Engagement for Medication aspects of pathway

|  | **CARIBOU-1 Pathway** | | | | **TAU** | | | **Comparing groups** |
| --- | --- | --- | --- | --- | --- | --- | --- | --- |
|  |  | **Clinician Adherence** | | **Participant Exposure** |  | **Participant Exposure** | | **(exposed relative to applicable)** |
| **Component** | **Main Component Participant** | **# applicable** | **# offered (% of applicable)** | **# youth engaged**  **(% of offered)** | **Main Component Participant** | **# applicable** | **# exposed (% of applicable)** | ***p* (Fisher’s exact)** |
| Fluoxetine offered as first-line, if not on antidepressant at baseline and no previous fluoxetine trial and moderate-severe depression, | Youth (N=35) | 21   - 3 had previous trials of fluoxetine - 4 already on sertraline at baseline with some evidence of response - 7 not on any medication in the pathway | 21 (100%) | 21 (100%) | Youth (N=31) | 24   - 3 had previous trials of fluoxetine - 3 on no medication throughout - 1 missing information | 16^a^ (68%)   - 4:sertraline^a^ - 1:fluvoxamine^a^ - 1:desvenlafaxine - 1:lamotrigine & mirtazapine^a^ - 1: lamotrigine & bupropion | 0.004 |
| If failed fluoxetine, sertraline offered as second-line. | Youth (N=35) | 3   - Escitalopram started by non-study psychiatrists during hospitalization for 2 additional participants | 3 (100%) | 3 (100%) | Youth (N=31) | 2 | 2 (100%) | 1 |
| If tolerated, medication continued until team review corresponding to “8 weeks since medication initiation” even if no response. | Youth (N=35) | 18 | 18 (100%) | 18 (100%) | N/C |  |  |  |
| If no response and not remitted at team review corresponding to “12 weeks since medication initiation”, discussion around switching medication. | Youth (N=35) | 6 | 6 (100%) | 6 (100%) | N/C |  |  |  |
| Not offered St. John’s Wort, Venlafaxine or Tricyclic Antidepressant | Youth (N=35) | 33 | 33 (100%) | 33 (100%) | N/C |  |  |  |

^a^each had comorbid anxiety disorder (i.e. generalized anxiety disorder, panic disorder or social anxiety disorder).

N/C= Not collected
